# Structural basis of vilazodone dual binding mode to the serotonin transporter

**DOI:** 10.21203/rs.3.rs-5671197/v1

**Published:** 2025-01-20

**Authors:** Iris E. Kalenderoglou, Andreas Nygaard, Caleb D. Vogt, Anton Turaev, Tillmann Pape, Nathan B.P. Adams, Amy Hauck Newman, Claus J. Loland

**Affiliations:** 1 Department of Neuroscience, Faculty of Health and Medical Sciences, University of Copenhagen, Copenhagen, Denmark; 2 Medicinal Chemistry Section, Molecular Targets and Medications Discovery Branch, National Institute on Drug Abuse – Intramural Research Program, National Institutes of Health, 333 Cassell Drive, Baltimore, MD 21224, United States; 3 NanoTemper Technologies, Floessegasse 4, 81369 Munich, Germany; 4 Structural Molecular Biology Group, Protein Structure & Function Program, Novo Nordisk Foundation Center for Protein Research, Faculty of Health and Medical Sciences, University of Copenhagen, Copenhagen, Denmark; 5 Core Facility for Integrated Microscopy (CFIM), Faculty of Health and Medical Sciences, University of Copenhagen, Copenhagen, Denmark

## Abstract

The serotonin transporter (SERT) plays a pivotal role in regulating serotonin (5-HT) signaling and is a key target in treating psychiatric disorders. SERT has a binding site (S1) for 5-HT that also serves as a high-affinity binding site for antidepressants. The antidepressant vilazodone has been shown to inhibit SERT by binding to an allosteric site. Here, we present the cryo-EM structure of SERT with vilazodone bound to the S1 site and extending towards the allosteric site. We systematically dissect the vilazodone molecule into fragments and find that the terminal indole ring is the key determinant for its high affinity to SERT. Further, unlike typical Na^+^-dependent SERT-selective antidepressants, vilazodone exhibits a dissociation constant (*K*_D_) for purified SERT in the nanomolar range both in the presence or absence of Na^+^. We substantiate this binding mode by exploring the conformational impact of vilazodone binding to SERT using site-specific insertion of the fluorescent non-canonical amino acid Anap. Our results offer novel molecular insight into the distinct pharmacological profile of a clinically used polymodal antidepressant.

## Introduction

Depression is one of the most prevalent neuropsychiatric disorders, affecting approximately 4.4% of the global population^1^. While SSRIs^2–6^ have superseded older antidepressants, they are not devoid of side effects^7^, which significantly reduce patients’ adherence to the treatment^5,8,9^. Vilazodone (Viibryd^®^), characterized as a selective serotonin (5-HT) reuptake inhibitor (SSRI) and a 5-HT_1A_ receptor partial agonist (SPARI)^10,11^, offers a more favorable profile with fewer instances of sexual dysfunction, weight gain, and emotional blunting^12–16^. It also exhibits a faster onset of action and higher efficacy^12–16^, making it a compelling antidepressant treatment.

The inhibition of SERT is a common therapeutic strategy for the treatment of neuropsychiatric disorders, including depression, anxiety, and post-traumatic stress disorder. SERT plays a crucial role in regulating the availability of 5-HT, a neurotransmitter involved in numerous brain functions, such as mood, sleep, appetite, and memory^17^. Located in the presynaptic neurons, SERT facilitates the reuptake of released 5-HT, thereby terminating neuronal signaling^17–19^. SERT is a Neurotransmitter Sodium Symporter (NSS) and belongs to the solute carrier (SLC) 6 family^20^, which is characterized by a common structural fold first found in the bacterial amino acid transporter LeuT^21^. This fold consists of two topological repeats, each of five transmembrane helices (TMs)^19,21^ that facilitate substrate transport through an alternating access mechanism^22–24^. It has been suggested that SERT regulates 5-HT availability through occupation of two substrate binding sites (S1 and S2)^25^. Specifically, S1 is composed of residues within TM1, TM3, TM6, and TM8, whereas the S2 site is situated in the extracellular vestibule recruiting residues from TM10 and TM12^25^. The functional role of the S2 site remains unknown. Electrophysiological studies have suggested that only one 5-HT molecule is transported along with one Na^+^ and one Cl^-^ ion per transport cycle^26^. After intracellular substrate release, the return-step is then believed to be facilitated by a counter-transport of either K^+^ or H^+26^, providing an overall electroneutral 5-HT transport cycle^27^.

The S1 site in SERT, accommodating substrates and typical inhibitors, can be organized into three compartments: subsite A (TM1, TM6, and TM8) is primarily engaging with the positively charged amino group of ligands (D98 for SERT), subsite B (TM3 and TM8) is crucial for hydrophobic coordination of the ligand’s aromatic group, and subsite C (TM6 and TM10) is proximal to the extracellular vestibule^28^. A key feature in the substrate translocation process is the formation of an electrostatic interaction between R104 and E493 located in the extracellular vestibule. This interaction is thought to be essential for the gating mechanism of SERT, which regulates the entry of solutes towards the S1 site^29,30^. Furthermore, close to the S1 site are two Na^+^ binding sites^31,32^. Studies have demonstrated that a Na^+^-rich environment promotes outward-open states of NSS proteins, facilitating extracellular 5-HT binding^33–37^. In contrast, in the presence of K^+^ ions, which are believed to be counter-transported^27,38^, the equilibrium shifts towards an inward-facing conformation^25,39,40^. Even though a K^+^ gradient is not required for 5-HT uptake, such a gradient drives the forward transport cycle^41^. All investigated SSRIs exhibit Na^+^-dependent binding and seem to stabilize an outward-open SERT conformation^25,31,42–46^.

The extracellular vestibule of SERT not only serves as an entry pathway for 5-HT and inhibitors binding to the S1 site but also possesses an allosteric binding site^31,45,47–49^. Our recent studies on vilazodone^45^ showed that it inhibited the dissociation of pre-bound [^3^H]imipramine with an allosteric potency of ~14 nM. The concomitant cryo-EM structure of the SERT-imipramine-vilazodone complex revealed that SERT was stabilized in the outward-open state with imipramine bound to the S1 site and vilazodone bound to an allosteric site, residing partly in the extracellular vestibule and partly within a cryptic site between TM11 and TM12. This aligned with studies using the substituted cysteine accessibility method^50^, in which vilazodone binding was suggested to stabilize an outward-open and inward-closed conformation^51^. The molecular interactions of vilazodone with SERT were also explored with modelling^53^, predicting vilazodone to bind as an orthosteric ligand with the benzofuran ring interacting with the S1 site residues and the indole group extending towards the extracellular vestibule. Interestingly, vilazodone’s inhibition of [^3^H]5-HT uptake revealed a Hill coefficient of ~1.8^45^, which suggested cooperativity between multiple inhibitor binding sites^52^. This prompted us to search for additional binding modes, which might be revealed in the absence of other supporting ligands, such as imipramine.

In the present study, we employ molecular pharmacology, cryo-EM, and fluorescence-based techniques to elucidate the molecular mechanism behind vilazodone binding to SERT. The cryo-EM structure shows one molecule of vilazodone bound at the S1 site extending towards the extracellular vestibule, thus revealing a different pose than the one determined previously. Utilizing spectral shift assays of purified and fluorescently labelled SERT, we demonstrate a vilazodone affinity in the low nM range, both in the presence of Na^+^ or K^+^. To further dissect the interaction dynamics, we systematically deconstructed the vilazodone molecule into its benzofuran and indole fragments. The indole analogues emerged as highly potent 5-HT reuptake inhibitors, whereas the benzofuran ligands exhibited low potency in this assay. Moreover, our engineered SERT construct labelled with the fluorescent non-canonical amino acid (ncAA) Anap^54^, suggested that vilazodone binds in both Na^+^ or K^+^ environments and indicated that vilazodone induced distinct SERT conformations depending on the ionic conditions. Overall, this work uncovers the ability of a prescribed antidepressant to adopt distinct high-affinity binding modes in SERT depending on the bound ion or ligand in the S1 site. These findings not only decode the intricate mechanism of SERT inhibition by vilazodone but also provide valuable insights into its unique pharmacological properties.

## Results

### Mutations in the allosteric site do not decrease the inhibition potency of vilazodone.

In our previous work^45^, we solved the cryo-EM structure of SERT in complex with imipramine and vilazodone, revealing the allosteric inhibition mechanism by vilazodone. Notably, the inhibition of [^3^H]5-HT uptake by vilazodone demonstrated a Hill coefficient of 1.8^45^. Given that this could be the result of cooperative binding of multiple vilazodone molecules, we aimed to further investigate its inhibition mechanism.

**Extended Data Fig. 1:**
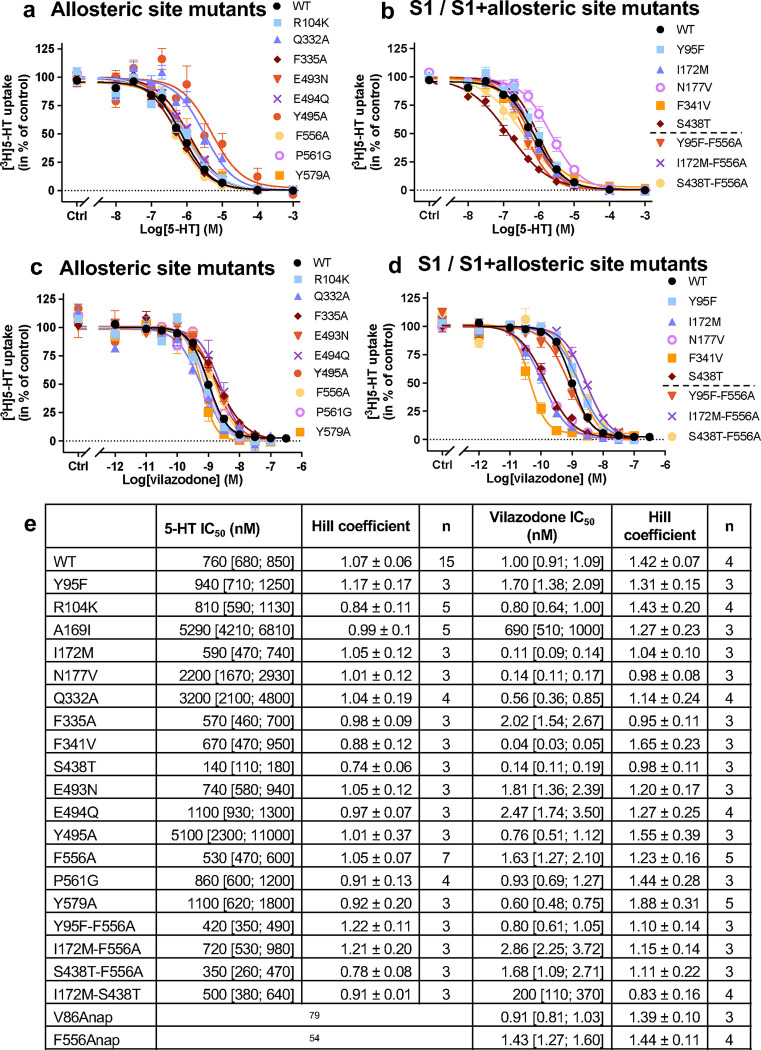
Effect of S1 and allosteric site mutants on IC_50_ for 5-HT and vilazodone. (**a**,**b**) Inhibition of [^3^H]5-HT uptake by increasing 5-HT concentrations on SERT WT and either (**a**) allosteric or (**b**) S1 or S1+allosteric site mutants. Similarly, in (**c**) and (**d**), inhibition of [^3^H]5-HT uptake by increasing vilazodone concentrations on SERT WT and allosteric or S1 and S1+allosteric site mutants, respectively. Experiments performed on intact COS-7 cells transiently transfected with SERT WT or mutants. Data are shown as mean ± S.E.M. (error bars) of n = 3–15 biological replicates performed in triplicates. (**e**) The inhibitory potency, Hill coefficient and “n” number of individual repeats of 5-HT and vilazodone [^3^H]5-HT uptake experiments. Data are shown as mean and [S.E. interval] calculated from pIC_50_ ± S.E. Hill coefficient error bars represent standard error of n = 3–15 values.

**Fig. 1:**
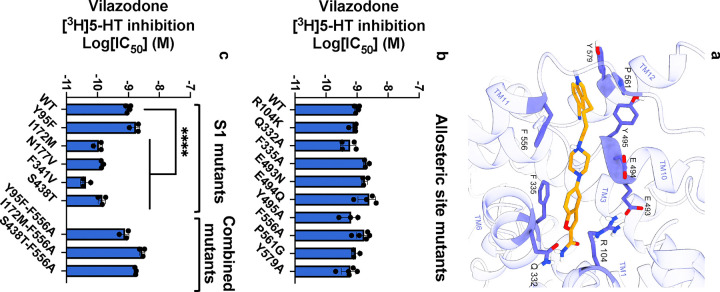
Effect of mutations in the S1 and the allosteric site on vilazodone affinity. **a,** A top view of allosterically bound vilazodone (orange) to SERT’s extracellular vestibule (purple ribbons) acquired from the previous cryo-EM structure^25^ (PDB ID: 7LWD). The interacting side chains are shown (purple)^25^. **b,c,** The half-maximal inhibitory concentration (IC_50_) of vilazodone for SERT WT and mutants located at the allosteric site (**b**), the S1 site, or the combined double mutants (**c**). The IC_50_ values are derived from whole-cell [^3^H]5-HT uptake competition experiments performed on intact COS-7 cells transiently expressing SERT WT or mutants. Mutations showed no significant change in the IC_50_ values (p > 0.9999) compared to SERT WT except from I172M, N177V, F341V and S438T showing significant change (****, p < 0.0001). The statistical analysis was performed using one-way ANOVA (95% CI, significance set at p < 0.05) with Dunnett multiple comparison correction. Data are shown as mean ± S.E.M. (error bars) of n = 3–5 biological replicates, performed in triplicates. Refer to Extended data Fig. 1 for IC_50_ curves, calculated IC_50_ values, and Hill coefficient values.

We hypothesized that if vilazodone binds to the allosteric site identified in the previous cryo-EM structure, then mutating the interacting residues would reduce its potency to inhibit [^3^H]5-HT transport. Accordingly, we mutated 9 residues (R104K, Q332A, F335A, E493N, E494Q, Y495A, F556A, P561G, and Y579A) predicted to be important for vilazodone binding (Fig. 1a). We expressed the SERT mutants in COS-7 cells and studied their effect on vilazodone binding potency (Fig. 1b, Extended Data Fig. 1). All mutants transported [^3^H]5-HT with similar potencies to that of SERT WT (Extended Data Fig. 1). Vilazodone inhibited [^3^H]5-HT transport by SERT WT with an IC_50_ around 1 nM, which agrees with our previous findings^45^. Surprisingly, all mutants showed no significant change in the IC_50_ values for vilazodone (p > 0.9999), ranging between 0.56–2.47 nM (Fig. 1b). Thus, these findings did not correlate with our previous study, where the same mutants had a pronounced effect on the allosteric potency of vilazodone^45^. However, these previously reported data were based on the ability of vilazodone to allosterically inhibit the dissociation of S1-bound [^3^H]imipramine. We reasoned that the presence of [^3^H]imipramine could potentially prevent vilazodone from assuming multiple binding poses and, therefore, the tested SERT mutants used here were affecting only one of the possible binding modes.

Molecular dynamics (MD) simulations have assessed how vilazodone binds in the absence of an S1 ligand^53^. The results suggested a different binding pose of vilazodone compared to the one in the SERT-imipramine-vilazodone structure^45^, with its benzofuran terminus occupying the S1 site and the indole terminus extending into the extracellular vestibule^53^. Hence, we designed a set of S1 mutants (Y95F, I172M, N177V, F341V, and S438T), which were predicted to interact with vilazodone in this alternative binding pose. Of note, several of these residues have been shown to decrease the affinity for a variety of SSRIs^45,55^. The 5-HT uptake inhibition of the S1 mutants resulted in potencies for vilazodone (Fig. 1c, Extended Data Fig. 1) that did not provide a clear indication of a specific vilazodone binding pose. The SERT mutants either did not significantly decrease the vilazodone potency or, in some cases, potentiated its inhibition to a significant extent (e.g., I172M, N177M, F341V, and S438T) (Fig. 1c). To further mechanistically explore the binding pose of vilazodone, we generated S1 mutants combined with the allosteric site mutant F556A (Y95F-F556A, I172M-F556A, and S438T-F556A), which were predicted to alter the cation-π interactions with the indole terminus. However, these mutants did not significantly change the inhibition potency of vilazodone either (Fig. 1c). Since our molecular pharmacology approach was unable to support the computationally predicted binding pose of vilazodone, we turned to cryo-EM to capture its interaction at an atomistic level.

### Cryo-EM reveals an orthosteric binding mechanism of vilazodone.

**Extended Data Fig. 2:**
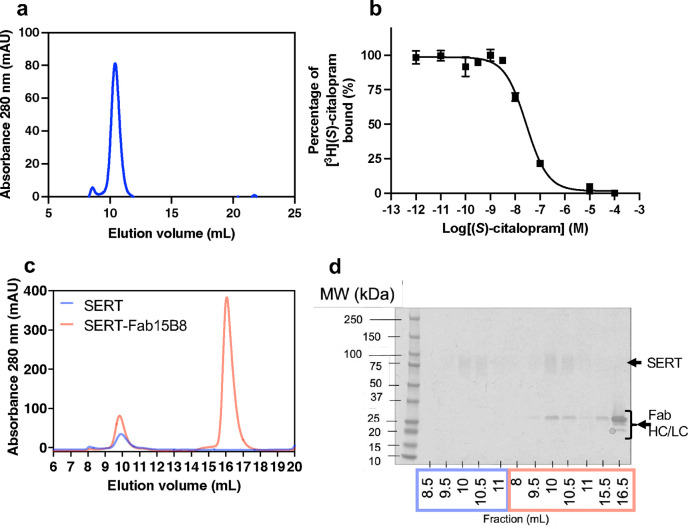
The SERT-Fab 15B8 complex with vilazodone was isolated in its active form and at a purity level suitable for cryo-EM. (**a**) Size exclusion chromatogram (SEC) demonstrating that SERT WT elutes at 10–12 mL fractions in a Superdex 200 increase column. (**b**) Scintillation proximity assay showing activity of SERT solubilized in GDN binds (*S*)-citalopram with a *K*_D_ = 17.7 [10.1; 28.4] nM in equilibrium binding homologous competition experiment. (**c**) Analytical SEC using vilazodone-bound SERT (blue) eluting at around 10 mL fractions and co-elution of vilazodone-bound SERT with Fab 15B8 (orange) at 10 mL with free F_ab_ 15B8 eluting at 16 mL. (**d**) Coomassie-stained SDS-PAGE of the SEC elution fractions shown in panel **c** demonstrating the glycosylated bands of SERT 75–100 kDa and Fab fragment chains at 20–25 kDa. In the second elution (orange) SERT was co-eluted with Fab.

**Extended Data Fig. 3:**
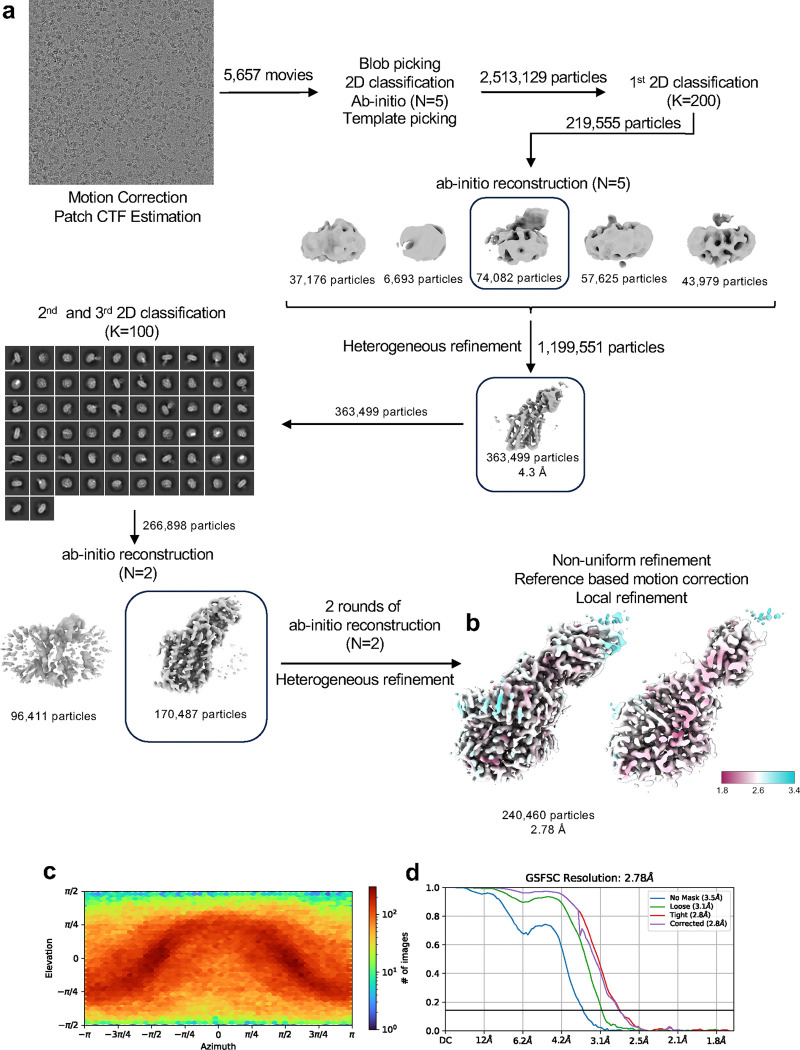
Structure determination using cryo-EM. (**a**) Workflow of cryo-EM data processing of SERT-Fab15B8-vilazodone complex in the outward-open conformation. The entire data process was performed using CryoSPARC^71^. Details can be found in Methods. (**b**) Local resolution of the sharpened maps shown as side view and longitudinal section view. (**c**) Angular distribution of the particles used for the final reconstruction. (**d**) Gold-standard FSC curves of the final non-uniform refinement. The final resolutions of the cryo-EM maps were determined by the gold-standard with a threshold of 0.143.

**Extended Data Fig. 4:**
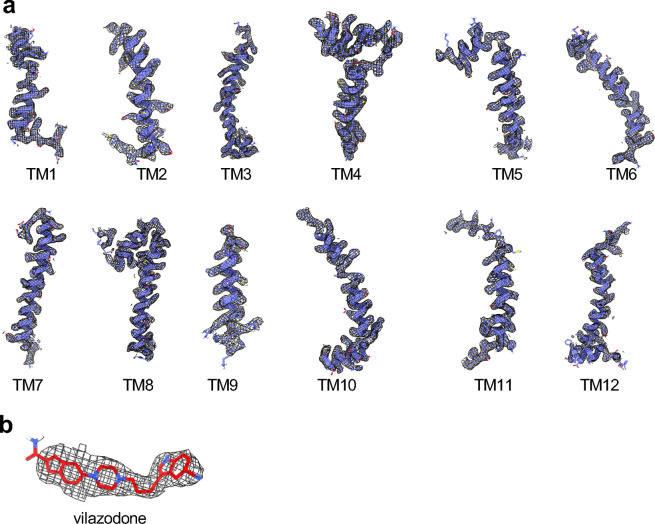
Representative EM densities of SERT and vilazodone. (**a**) Representative EM maps of SERT TMs and EM densities, contoured at 6.2 σ. (**b**) EM map of fitted vilazodone (red sticks).

**Extended Data Fig. 5:**
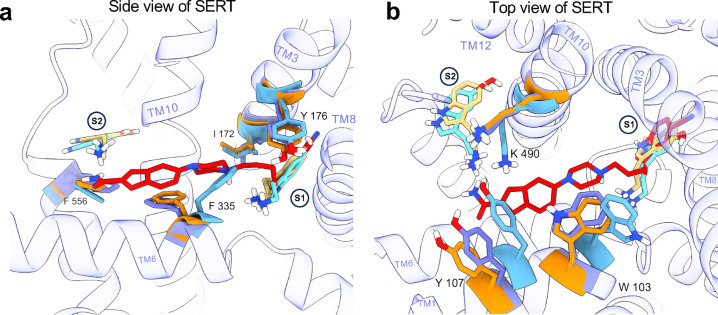
Comparison of 5-HT-bound and vilazodone bound structures reveal common interaction patterns. (**a**) Side view comparison of the 5-HT-bound outward-open (PDB ID: 7LIA) – (5-HT in yellow, SERT in orange), the 5-HT-bound occluded (PDB ID: 7MGW) – (5-HT in cyan, SERT in light blue), and the vilazodone-bound – (vilazodone in red, SERT in purple), SERT structures. Structures are superimposed based on the Cα-carbons in the TMs, excluding the loops. (**b**) Top view of Panel (**a**). Comparing the hydrophobic gate and residues K490, Y107, and W103 to the 5-HT-bound structures, the vilazodone-bound SERT adopts an outward-open conformation.

**Fig. 2:**
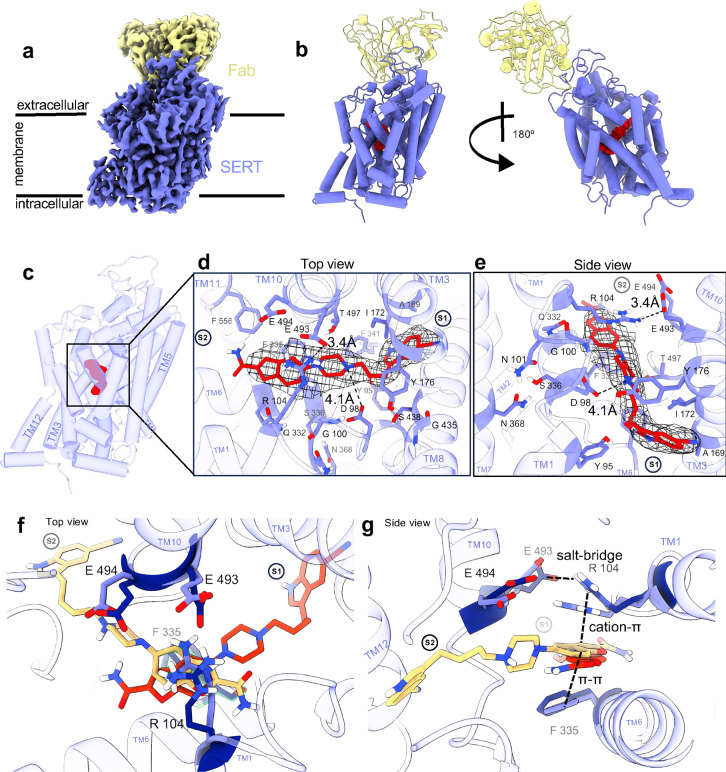
Structure of SERT-Fab with vilazodone bound in the S1 site. **a**, Cryo-EM density (2.78 Å) of SERT (purple) in complex with the Fab 15B8 fragment (yellow). Estimated positions of membrane phospholipids surrounding SERT are illustrated with black lines. **b,** The fitted structure of SERT-Fab (purple tube helices) and turned 180° (right). Vilazodone (red spheres) was modeled into a non-protein electron density in the S1 site. **c,** The fitted SERT structure (purple) in 30% transparency, marking the electron density of vilazodone (red). **d,** Zoomed-in top view of vilazodone (red sticks) with depicted stabilizing side chain and backbone residues (purple). The electron density for fitting vilazodone is shown as black mesh. **e,** Side-view of vilazodone binding with SERT stabilizing residues. The S2 site is faded in the background. **f,g,** Superimposition of the vilazodone complexes in the presence of imipramine (PDB: 7LWD, yellow) and the structure solved herein (red). The constellation of centrally interacting side chain residues shown in dark blue and purple respectively.

To obtain the cryo-EM structure of the SERT-vilazodone complex in the absence of imipramine, we expressed the full-length SERT WT in Expi293 mammalian cells using the BacMam transduction system^56^. SERT was solubilized in a glyco-diosgenin (GDN) buffer and purified using a C-terminal twin-strep tag (Extended Data Fig. 2). Notably, GDN-solubilized SERT showed comparable (*S*)-citalopram binding properties (*K*_D_ = 17.7 [10.1; 28.4] nM, Extended Data Fig. 2) as for SERT expressed in membranes (~5 nM^45^). Vilazodone (10 to 100 μΜ) was present throughout the purification and grid preparation stages. The 15B8 Fab fragment served as a fiducial for cryo-EM facilitating the precise alignment of micelle-embedded SERT particles (Fig. 2a,b). As previous findings have shown, the co-eluted 15B8 Fab-bound SERT (Extended Data Fig. 2) has the capacity to undergo all the conformational transitions essential for 5-HT transport^25,40,57,58^, allowing us to hypothesize a vilazodone-induced SERT conformation within this spectrum. We successfully determined the structure of the SERT-vilazodone assembly at a global resolution of 2.78 Å (PDB ID: 9HCO, Extended Data Fig. 3,4). GDN-solubilized SERT exhibited an outward-open conformation with the extracellular vestibule hydrated and a densely packed intracellular side. The N-terminus (residues 1–76) and C-terminal purification tags are not resolved. The Root Mean Square Deviations (RMSDs) of the backbone Cα atoms of the TM helices between this structure and the SERT structures with imipramine-vilazodone, 5-HT outward-open, 5-HT occluded, and ibogaine inward-open structures are 0.64 Å, 0.70 Å, 1.70 Å, and 3.89 Å, respectively. Moreover, TM1 does not adopt the characteristic kink of the inward-facing ibogaine- or 5-HT-bound conformations, signifying that the intracellular gate of SERT is closed (Fig. 2c)^25,40^. Furthermore, superimposing the Cα in the TMs with those of the 5-HT-bound outward-open (PDB ID: 7LIA)^25^ and -occluded (PDB ID: 7MGW)^25^ structures of SERT suggests that the conformations of the key residues F335, Y107, and W103 correlate with an open extracellular gate. With the above observations, we can conclude that the vilazodone-bound SERT structure is in the outward-open structure (Extended Data Fig. 5).

Within the cryo-EM density, we identified a non- proteinaceous electron density at the S1 site, extending into the vestibule towards, but not reaching, the S2 site (Fig. 2c-e). We attributed this density to a vilazodone molecule with its indole moiety occupying the S1 pocket, in the same manner as S1-bound 5-HT (PDB ID: 7LIA), and with the benzofuran group facing the extracellular vestibule (Fig. 2d,e, Extended Data Fig. 5). Similar to other SSRIs^31,40,44–46^ and 5-HT^25^ in outward-facing SERT structures, the protonated amine in the piperazine group preserves its interaction with the carboxyl group of D98 positioned 4.1 Å apart (Fig. 2d). Furthermore, S336 participates in a hydrogen-bonding network with N368, which in turn forms a hydrogen bond with N101, approximately 4.5 Å from the non-proteinaceous density in the known chloride binding site ^31,40,42,44–46,59^. Nestled within subsite B, the indole ring of vilazodone is aligned between TM3 and TM8 through hydrophobic interactions with A169, I172, and Y95, which is blocking the extracellular side right above the indole group. In addition, Y176 is forming a stabilizing bridge between TM3 and TM8 through an aromatic hydrogen bond with the backbone carbonyl group of G435. One of the most prominent interactions is the π-π stacking between the indole ring and F341, whose sidechain is lifted upwards, filling the void below vilazodone. With this F341 conformation, vilazodone closely resembles SERT binding of sertraline and fluvoxamine^46^ but not paroxetine or (*S*)-citalopram^31^. Furthermore, the benzofuran group is interacting through π-π stacking with F335 and is stabilized by aromatic hydrogen bonding with the nearby Q332. Right above the benzofuran group, E493 projects towards the extracellular side where it forms a salt-bridge with R104 with a 3.4 Å distance. This putative salt-bridge (R104-E493) has been characterized as an essential intramolecular interaction signaling the opening and the occlusion of SERT. R104 also plays a pivotal role in the accommodation of allosteric ligands^48^. When vilazodone binds at the S1 site its benzofuran group forms potential cation-π interactions with R104, an interaction that is unique among all the investigated SSRIs. Although vilazodone does not reach F556 in the extracellular vestibule, the F556 phenyl is facing towards the water bulk, edge-to-edge with F335, similar to the paroxetine-bound conformation^31^. Since vilazodone likely binds with high affinity to both the S1 and allosteric sites, we superimposed the respective SERT structures according to the Cα of the TMs (PDB ID: 7LWD^45^) to compare the two vilazodone poses (Fig. 2f,g). Interestingly, the two vilazodone poses share the interaction with R104, suggesting that this region functions as a binding pocket that can accommodate a bulkier aromatic system, such as a benzofuran or an indole group. This comparison underscores the versatility of the binding pocket beneath R104.

**Fig. 3:**
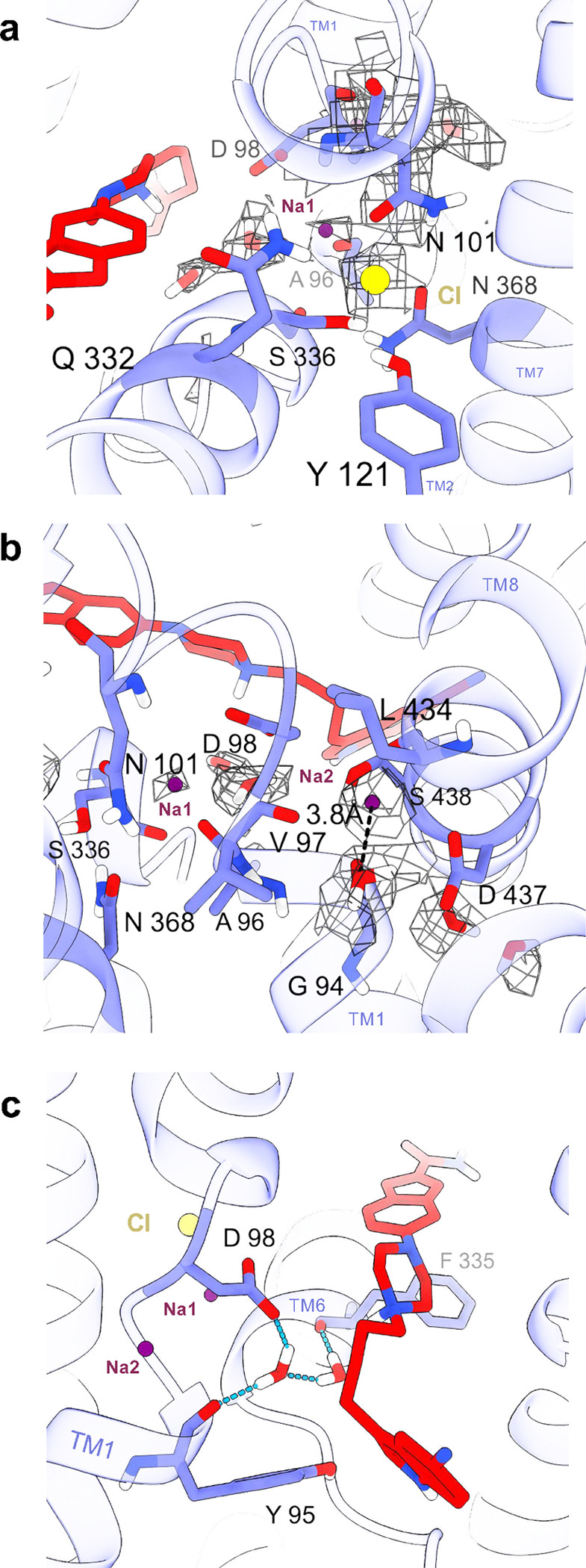
Structure of binding sites for ions and water. **a,** Side view of SERT showing the binding sites for Na1 (magenta sphere) and Cl (yellow sphere). The side chains forming the sites are shown (purple sticks). The EM density for ions and N101 are shown as mesh. The EM density of N101 is shown as a reference level corresponding to the density of SERT. Vilazodone is partly shown (red sticks). **b,** Side view of SERT showing Na1 and Na2 with the EM density as mesh. Resolved water molecules with EM density (mesh) and interacting residues are shown. Vilazodone is in red sticks. **c,** Side view of SERT showing the hydrogen bonding network between the water molecules and Y95, D98, and F335. Hydrogen bonds, Na1 and Na2, chloride, and vilazodone are shown.

We identified the ion binding sites where the Na2 and Cl ions were well resolved and coordinated as seen previously^31,46,59^ (Fig. 3a,b). A water molecule was also identified^31^ at a 3.8 Å distance from the Na^+^ ion in the Na2 site (Fig. 3b), forming a hydrogen bond with D437 that further stabilizes the Na2 cavity. In contrast, the presence and occupancy of the Na1 site is more questionable. We resolved the N101 side chain and encountered a weak density extending towards A96 (Fig. 3a,b). This density can potentially accommodate a Na^+^ ion surrounded by weak stabilizing interactions from the A96 backbone carbonyl (1.8 Å), N101 carbonyl (3.2 Å), D98 carboxyl (3.2 Å), S336 hydroxyl (3.6 Å), N368 carbonyl (4.0 Å), and the water molecule within 4.3 Å distance. The discerned density is within the Na1 binding pocket determined in previous SERT cryo-EM structures^25,32^. Between the piperazine moiety and the Na1 site, where the EM density has a resolution below 2.4 Å, two potential water molecules are revealed intricately connected by hydrogen bonds, forming a cooperative network with the F335 and Y95 backbones, as well as the D98 sidechain (Fig. 3c).

**Fig. 4:**
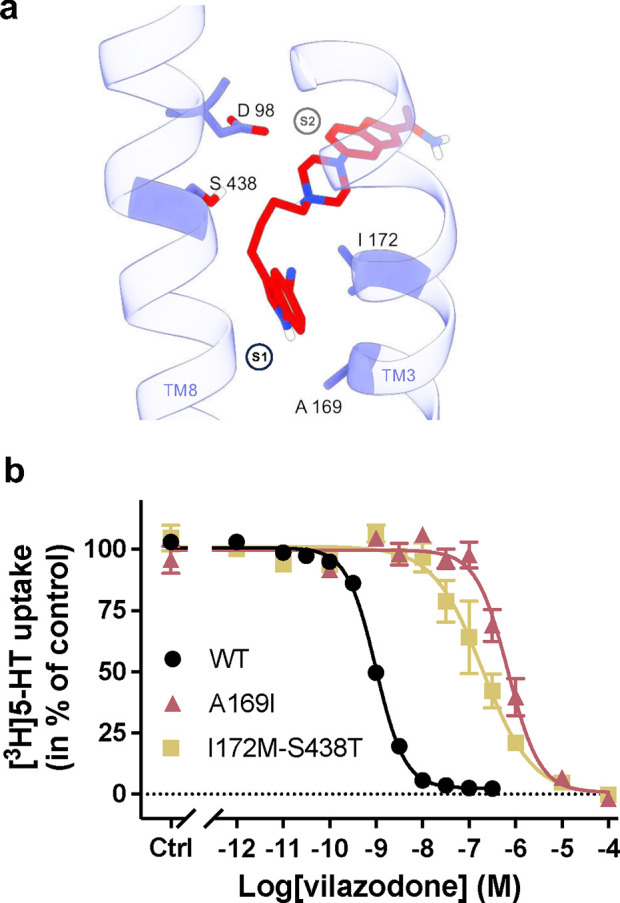
Key SERT residues involved in the inhibition of SERT by vilazodone. **a,** Side view of the TM3 and 8 key residues (D98, A169, I172, S438) forming the vilazodone binding site. **b,** Measurement of the potency of vilazodone inhibition for SERT WT (black), A169I (red) or I172M-S438T (yellow) mutants. Inhibition potencies were calculated equal to A169I: 690 [510; 1000] nM and I172M-S438T: 200 [110; 370] nM. The two SERT mutants showed significant changes in the IC_50_ values (****, p < 0.0001) compared to SERT WT. The statistical analysis was performed using one-way ANOVA (95% CI, significance set at p < 0.05), with Dunnett multiple comparison correction. Data are shown as mean ± S.E.M. (error bars) of n = 3–4 biological replicates, performed in triplicates. Refer to Extended data Fig. 1 for IC_50_ curves and calculated IC_50_ and Hill coefficient values.

The cryo-EM structure allowed us to conduct a deeper structure-guided analysis of the SERT-vilazodone complex with the purpose of creating SERT mutants that would challenge vilazodone inhibition potency while not perturbing 5-HT uptake. Particularly, the aliphatic chain connecting the piperazine with the indole group was lying in the vicinity of I172 and S438 (Fig. 4a). Thus, upon screening interacting residues, we generated the SERT I172M-S438T double mutant and expressed it in COS-7 cells. We found that vilazodone exhibited a significant 200-fold decrease in the [^3^H]5-HT uptake inhibition potency relative to that for SERT WT (Fig. 4b, Extended Data Fig. 1e). Furthermore, we observed that the indole group of vilazodone was coordinated by A169 in TM3 (Fig. 4a). To explore this further, we made the SERT A169I mutant, reasoning that the bulkier isoleucine might interfere with the stabilization of the indole group of vilazodone. Notably, A169 mutants have also been demonstrated to interfere with paroxetine^60^ and vortioxetine binding^61^. As predicted, vilazodone inhibition of [^3^H]5-HT uptake was decreased significantly by nearly 700-fold for SERT A169I with an IC_50_ of 690 [510; 1000] nM (Fig. 4b, Extended Data Fig. 1e).

### SERT binds vilazodone Na^+^-independently with nM affinity.

**Extended Data Fig. 6:**
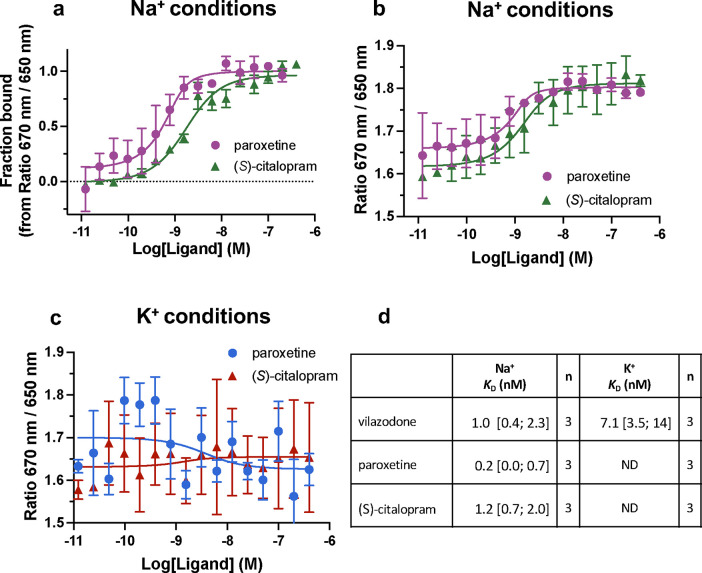
Control experiments for the fluorescence equilibrium binding assays using paroxetine and (*S*)-citalopram. (**a**) Paroxetine (magenta circles) and (*S*)-citalopram (green triangles) binding to SERT coupled with 2^nd^ generation NHS dye (NanoTemper Technologies GmbH, Munich, Germany) in 150 mM Na^+^ buffer using spectral shift binding isotherm assay at ratio between 670 and 650 nm (fraction bound) using a Dianthus (NanoTemper Technologies GmbH, Munich, Germany) instrument. (**b**) Binding isotherm (measured ratio of 670 nm / 650 nm) for paroxetine (magenta circles) and (*S*)-citalopram (green triangles) binding to SERT. Bottom and top plateaus were normalized to 0.0 (no ligand binding) and 1.0 (100 % ligand-bound SERT) fraction resulting in Panel (**a**). (**c**) Similar to (**b**), binding isotherm (measured ratio of 670 nm / 650 nm) for paroxetine (blue circles) and (*S*)-citalopram (red triangles) dye in 150 mM K^+^ buffer binding to SERT showing no detectable binding phenomena. (**d**) Equilibrium dissociation constants *K*_D_ acquired by Spectral Shift. Data in (**a-c**)are shown as mean ± S.E.M. (error bars) and in (**d**) as mean and [S.E. interval] of n = 3 biological replicates, performed in triplicates.

**Fig. 5:**
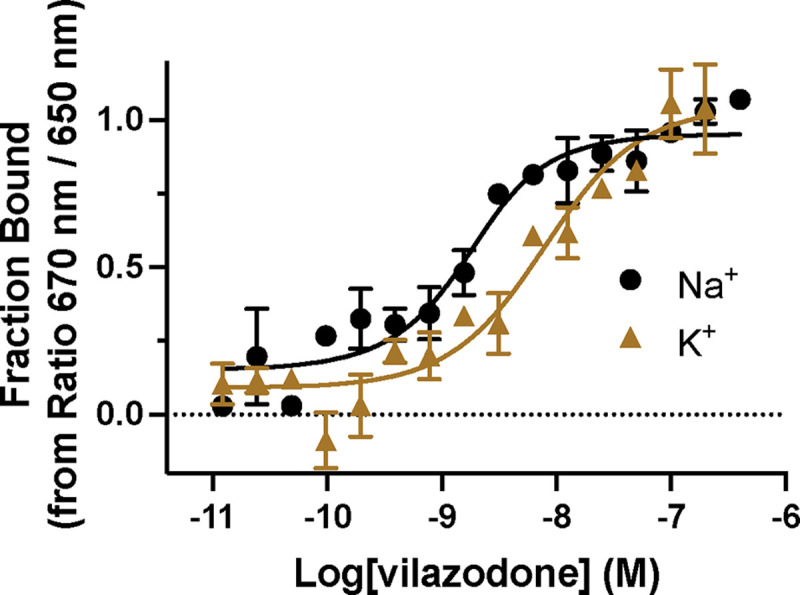
Fluorescence equilibrium binding show distinct vilazodone affinities in Na^+^ and K^+^. Vilazodone binding to SERT coupled with NHS dye in either 150 mM Na^+^(black circles) or K^+^(brown triangles) buffer using spectral shift binding isotherm assay at ratio between 670 and 650 nm (fraction bound). Data are shown as mean ± S.E.M. (error bars) of n = 3 biological replicates, performed in triplicates. Refer to Extended data Fig. 6 for control experiments and calculated *K*_D_ values.

Given the unique binding pose of vilazodone and the observation that the Na1 site might be collapsed in our cryo-EM structure, we next investigated whether vilazodone binding was ion-dependent. To this end, we used Spectral Shift assays^62^ to directly determine the vilazodone affinity for purified SERT in Na^+^- and K^+^-rich environments. Spectral shift relies on fluorescence from a dye coupled to SERT purified in detergent micelles. To confirm that our labeled SERT is sensitive to vilazodone binding, we tested for a ligand-induced spectral shift in the emission spectrum under isothermal conditions, which we measured using a Dianthus instrument^62^ (See Methods). Accordingly, to fluorescently labeled SERT we titrated vilazodone in a buffer containing either 150 mM NaCl or KCl. The *K*_D_ values for vilazodone in Na^+^ and K^+^ were measured to be 1.0 [0.4; 2.3] nM and 7.1 [3.5; 14] nM, respectively (Fig. 5). In comparison, the *K*_D_ values for (*S*)-citalopram and paroxetine in Na^+^ were 0.2 [0.0; 0.7] nM and 1.2 [0.7; 2.0] nM, respectively (Extended Data Fig. 6a,b,d), agreeing with previous literature findings^31^. We were unable to detect any binding in K^+^, underscoring the Na^+^-dependency for the binding of these two conventional SSRIs (Extended Data Fig. 6c,d).

**Extended Data Fig. 7:**
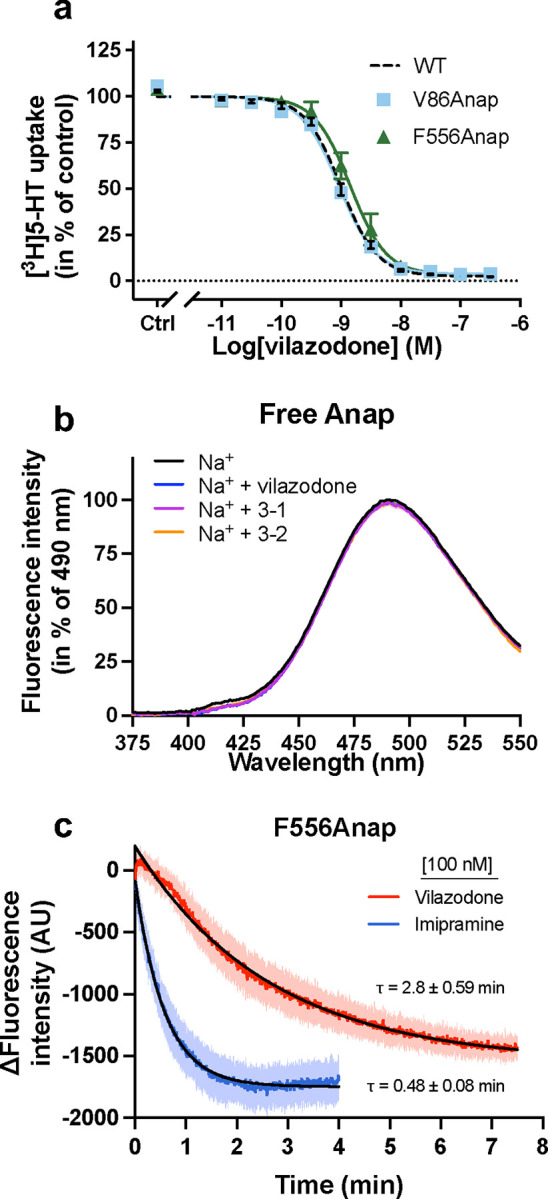
Effects of vilazodone on SERT function and Anap fluorescence. (**a**) [^3^H]5-HT uptake for SERT WT (black dotted line), V86Anap (blue squares) and F556Anap (green triangles) following pre-incubation with increasing concentrations of vilazodone. Data are normalized to the uptake obtained in the absence of vilazodone (control) and fitted by a non-linear regression. The IC_50_ values for V86Anap and F556Anap of 0.91 [0.84; 0.99] nM and 1.42 [1.25; 1.62] nM, respectively, are not significantly different (V86Anap: p = 0.79; F556Anap: p = 0.07) from that of WT (0.99 [0.91; 1.09] nM). The statistical analysis was performed using a using one-way ANOVA with Dunnett multiple comparison correction. (**b**) Fluorescence emission spectra (360 nm excitation) of 5 nM free Anap incubated in Na^+^ without (black) and with vilazodone (blue), CDV-3–1 (magenta) or CDV-3–2 (orange). Data are normalized to the fluorescence intensity at λ_max_ (490 nm). Error bars and error envelopes are mean ± S.E.M, n = 3–4. (**c**) Real-time changes in fluorescence intensity (arbitrary units) at 450 nm for SERT F556Anap pre-equilibrated in 200 mM Na^+^. Changes are recorded following the application of 100 nM vilazodone (red) or imipramine (blue). Data are normalized to the fluorescence intensity at time 0 and modelled by a one-phase exponential regression. Error envelopes and τ values are mean ± S.E.M., n = 3–5.

**Fig. 6:**
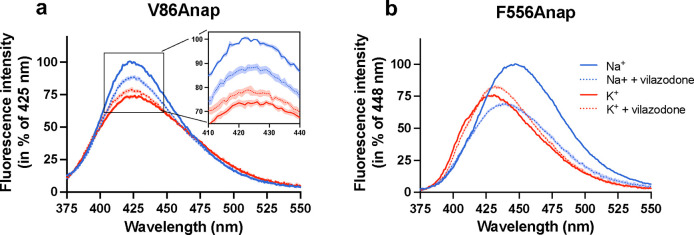
Vilazodone induces distinct conformational dynamics in Na^+^ and K^+^. **a**,**b**, Fluorescence emission spectra of purified SERT V86Anap (**a**) or F556Anap (**b**) excited at 360 nm. Spectra are recorded following incubation in 200 mM Na^+^ (blue) or K^+^ (red) with (dashed line) or without (solid line) 200 nM vilazodone. Color legends for **a** are the same as **b**. Data are normalized to the fluorescence intensities at λmax in Na^+^ (V86Anap: 425 nm; F556Anap: 448 nm). Data are shown as mean ± S.E.M. (error envelopes) of n = 3–5 biological replicates.

The observation that vilazodone may bind in the presence of both Na^+^ and K^+^ suggests either that vilazodone binds to both the SERT conformational states induced by Na^+^ and K^+^, respectively, or that vilazodone *per se* binds ion-independently. To distinguish between these possibilities, we investigated whether vilazodone binding had any impact on the conformation of SERT by inserting the fluorescent ncAA Anap at position V86 in TM1a (V86Anap) or F556 in TM11 (F556Anap), on the intracellular or extracellular sides, respectively. Detecting changes in Anap fluorescence as a conformational reporter has previously enabled us to show changes in SERT conformational dynamics upon the binding of a set of ions and ligands^54^. Similarly, we used the Anap mutants to investigate structural changes induced by the binding of vilazodone in combination with either Na^+^ or K^+^. Notably, the potencies for vilazodone inhibition of [^3^H]5-HT uptake for these mutants were not significantly different from that for SERT WT (Extended Data Fig. 7a). We measured the fluorescent properties of the mutants, following incubation in the absence or presence of a saturating concentration of vilazodone (200 nM) in either Na^+^ or K^+^ conditions (Fig. 6). Both V86Anap and F556Anap demonstrated different fluorescence patterns in Na^+^ and K^+^, consistent with our previous findings, and likely reflecting the different conformational states adopted with the two ions^54^ (Fig. 6). In the presence of vilazodone, both V86Anap and F556Anap yielded changes in their fluorescence spectra. For V86Anap (Fig. 6a), which captures structural changes in the microenvironment around TM1a, the addition of vilazodone decreased the fluorescence in Na^+^ while increasing the fluorescence in K^+^. These results could indicate that vilazodone binds to SERT in both ionic conditions. For F556Anap, which has Anap inserted into the extracellular vestibule of SERT, the emission spectra with vilazodone and Na^+^ showed a large decrease in intensity accompanied by a blue-shift relative to that with Na^+^ alone (Fig. 6b). In contrast, vilazodone in K^+^ produced a red-shifted and increase in fluorescence compared to that of F556Anap with just K^+^. Given that vilazodone has no effect on the fluorescence of free Anap (Extended Data Fig. 7b), the observation that vilazodone changed the fluorescence of V86Anap and F556Anap in both Na^+^ and K^+^ suggests that vilazodone binds in both ionic conditions. Furthermore, the spectral differences between the vilazodone-bound mutants in Na^+^ and K^+^ indicate that the SERT-vilazodone complexes in Na^+^ and K^+^ are not the same.

Interestingly, the size of the spectral changes induced by binding of vilazodone for F556Anap in Na^+^ was sufficiently large for us to measure the real-time changes in fluorescence associated with the addition of vilazodone. For this experiment, we applied 100 nM vilazodone to F556Anap pre-equilibrated in Na^+^ and compared the time-dependent changes in fluorescence to those induced by the addition of 100 nM imipramine, shown previously to also decrease the fluorescence^54^ (Extended Data Fig. 7c). For both compounds, we observed time-dependent decreases in fluorescence well modelled by mono-exponential time courses. Although using a single concentration of ligand does not allow us to resolve their kinetic parameters, the traces show that the application of vilazodone, despite its higher SERT affinity, produces a 5.8-fold slower change in fluorescence compared to that of imipramine. This may indicate that vilazodone displays kinetic binding properties unlike those of the more classical SERT inhibitors that exclusively bind to S1.

### Deconstruction of vilazodone reveals moieties determining its affinity.

**Fig. 7:**
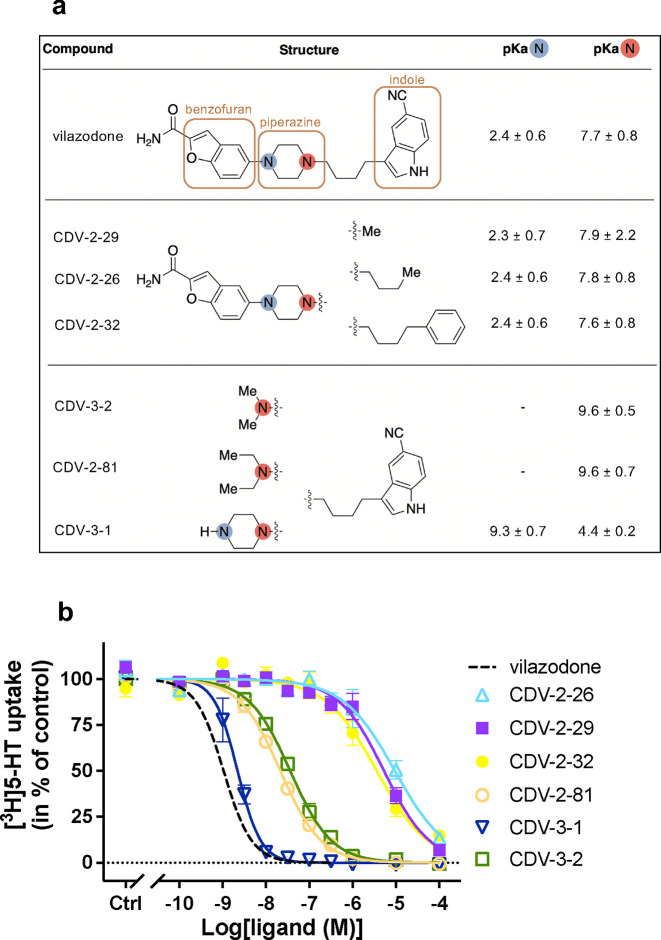
Vilazodone fragments reveal structural basis for SERT inhibition potency. **a,** The structures of vilazodone and the investigated fragments. The benzofuran, piperazine and indole groups are highlighted with brown boxes. The calculated pKa values included for the two piperazine N-atoms are listed. **b,** The inhibitory potency of the vilazodone fragments shown in **(a)** for SERT WT. Data are shown as mean ± S.E.M. (error bars) of n = 3–4 biological replicates, performed in triplicates. Refer to Extended data Fig. 1 for IC_50_ curves and calculated IC_50_ and Hill coefficient values.

The chemical structure of vilazodone is characterized by its elongated shape, featuring a 2-amido-substituted-benzofuran ring attached to a piperazine, which in turn is linked to an indole through a four-carbon chain. To elucidate the individual contribution of these moieties to the affinity for SERT, we dissected vilazodone into two different series of fragments (Fig. 7a). The first set of compounds retained the benzofuran and piperazine rings found in the parent structure while replacing the indole and four-carbon linker with a methyl (CDV-2–29), *n*-butyl (CDV-2–26), or *n*-butylbenzene (CDV-2–32). The second set of compounds preserved the indole and linking chain, removing the benzofuran (CDV-3–1) and deconstructing the piperazine ring (CDV-2–81 and CDV-3–2).

**Extended Data Fig. 8:**
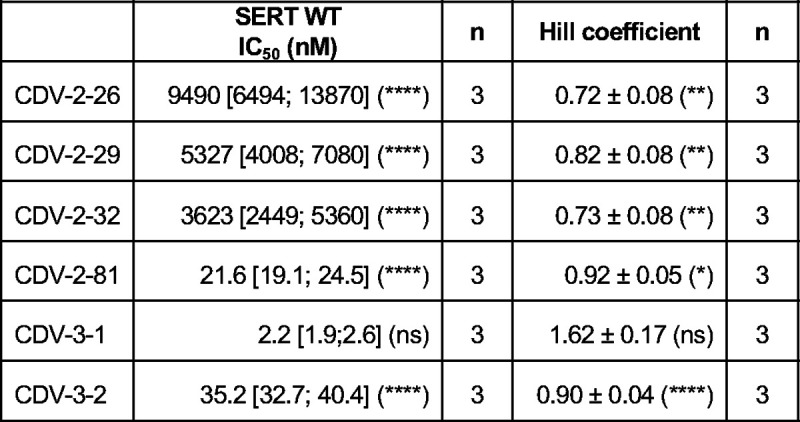
The inhibitory potency (IC_50_), Hill coefficient and “n” number of individual repeats of the vilazodone fragments [^3^H]5-HT uptake experiments. Data are shown as mean and [S.E. interval] calculated from pIC_50_ ± S.E. of n = 3 biological replicates, performed in triplicates. IC_50_ and Hill coefficient data were statistically analyzed with a Dunnett (IC_50_) and a Tukey (Hill coefficient) multiple comparison test-corrected one-way ANOVA (95% CI, significance set at p < 0.05). *, p <0.05; **, p <0.01; ***, p < 0.001; ****, p < 0.0001 represent significance levels from a Tukey multiple comparison test-corrected one-way ANOVA, comparing the mean to that of the vilazodone. Hill coefficient error bars represent standard error of n = 3 values.

When screened for their ability to inhibit [^3^H]5-HT uptake, fragments CDV-2–29, CDV-2–26, and CDV-2–32, which all contained the benzofuran, exhibited IC_50_ values in the μΜ range: 5300 [4000; 7100] nM, 9500 [6500; 14000] nM, and 3600 [2500; 5400] nM, respectively (Fig. 7b, Extended Data Fig. 8). Based on our vilazodone-bound structure, the benzofuran preserves its binding site in the pocket adjacent to R104 allowing cation-π interactions, which is essential for other allosteric ligands, such as Lu AF88273^48^. The comparison between the S1 and allosterically bound vilazodone structures (Fig. 2f,g) suggests that there might be a benzofuran binding hot-spot below the R104-E493 salt-bridge, essential for the high allosteric potency of vilazodone. However, all three benzofuran derivatives that were no longer tethered to the terminal indole lost affinity for SERT by several orders of magnitude compared to vilazodone. Notably, CDV-2–26 and CDV-2–32 contain a linker attached to the piperazine moiety to mimic the elongated structure of vilazodone, but these two fragments maintained their IC_50_ values in the μM range.

In contrast, indole-based fragments CDV-2–81, CDV-3–1 and CDV-3–2 (Fig. 7b), displayed orders of magnitude higher SERT inhibition potencies with IC_50_ values of 21.6 [19.1;24.5] nM, 2.2 [1.9; 2.6] nM, and 35.2 [32.7; 40.4] nM, respectively. Interestingly, the combination of the indole and piperazine (CDV-3–1) produced a similar IC_50_ value compared to vilazodone with only a 2.2-fold increase in [^3^H]5-HT uptake inhibition (Fig. 7b). These results suggest that the protonatable nitrogen of the piperazine ring increases the inhibition potency by at least an order of magnitude when combined with an indole (CDV-3–1) but not a benzofuran (CDV-2–26, CDV-2–29, and CDV-2–32). For each fragment, we calculated the pKa of the piperazine nitrogen using Epik^63^ to predict their protonation states and further locate the positive charge in reference to D98 that was seen adjacent to vilazodone in the cryo-EM structure. Based on these calculations, the piperazine in CDV-3–1 is protonated at a different nitrogen compared to vilazodone, placing it at the terminal secondary amine. The charge is further away from D98 (6.4 Å vs 4.1 Å). The similar IC_50_ values for vilazodone and CDV-3–1 could either suggest that the protonated nitrogen has minimal contribution to the affinity of vilazodone or that the changed protonation of CDV-3–2 could largely substitute for that of vilazodone. Notably, CDV-3–1 was the only fragment with a high Hill coefficient of approximately 1.62, not significantly different from that of vilazodone (Extended Data Fig. 8). Upon removal of the piperazine group (CDV-2–81 and CDV-3–2), the IC_50_ increases to 21.6 nM and 35.2 nM, respectively, demonstrating the importance of the indole terminus.

**Extended Data Fig. 9:**
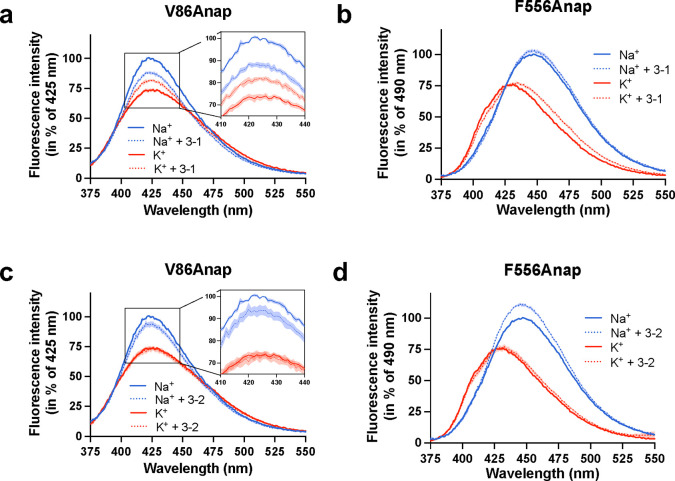
Fragments of vilazodone show different binding modes. **a,b,c,d,** Emission spectra of SERT V86Anap (**a,c**) or F556Anap (**b,d**) following incubation in Na^+^ (blue) or K^+^ (red) with (dashed line) or without (solid line) 200 nM CDV-3–1 (**a,b**) or CDV-3–2 (**c,d**). Data are normalized to the fluorescence intensities at λ_max_ in Na^+^ (V86Anap: 425 nm; F556Anap: 448 nm). Error envelopes are mean ± S.E.M., n = 3–5.

To assess whether these fragments, like vilazodone, could shift the conformation of SERT independently of the ions present, we recorded emission spectra of V86Anap and F556Anap. This was performed after incubation with CDV-3–1 and CDV-3–2 (which lacks the piperazine) in the presence of Na^+^ or K^+^ (Extended Data Fig. 9). In V86Anap experiments, CDV-3–1 shifted fluorescence in both ion environments similar to vilazodone, suggesting that CDV-3–1 binds in both Na^+^ or K^+^ (Extended Data Fig. 9a). The changes in fluorescence for F556Anap with CDV-3–1, on the other hand, were less pronounced (Extended Data Fig. 9b). Interestingly, the addition of CDV-3–2 gave rise to changes in fluorescence for both Anap SERT constructs in Na^+^ but not in K^+^ (Extended Data Fig. 9c,d), suggesting either that CDV-3–2 binding is Na^+^-dependent or that the state induced by its binding is very similar to the K^+^-bound SERT conformation. Notably, the smaller fluorescence changes induced by these two fragments compared to those of vilazodone could be attributed to the fact that its benzofuran ring is in close proximity to the F556Anap residue and, thereby, may impact on the local chemical environment of Anap through direct interactions.

## Discussion

Vilazodone is classified as an SSRI with its therapeutic properties primarily attributed to interactions with SERT and 5-HT_1A_ receptors. Multiple mechanistic factors likely contribute to the unique pharmacological profile of vilazodone. In our previous work^45^, we demonstrated that vilazodone acts as a non-competitive inhibitor of SERT and binds with high affinity to a pocket in the extracellular vestibule, supporting an allosteric mechanism of action. Notably, vilazodone exhibited a Hill coefficient of ~1.8, suggesting positive cooperativity for inhibiting SERT despite only a single density for vilazodone seen in the EM map^45^. This discrepancy, along with its distinct pharmacological profile, made us reason that potentially the bound imipramine biased the vilazodone binding pose in the cryo-EM structure. Accordingly, it inspired us to further investigate the structure-activity relationships of vilazodone for SERT inhibition and understand the molecular basis of this drug’s possible multimodal effect.

Compared to our previous findings, we report herein a cryo-EM structure of SERT, showing that in the absence of imipramine, vilazodone itself acts as an orthosteric inhibitor of SERT. We did not observe any additional EM densities that could be attributed to allosteric binding. The observed orthosteric binding could explain the unchanged vilazodone affinity when placing mutations in the allosteric site. However, if the S1 site was the only site available for vilazodone, we would have expected an impact on affinity from the S1 mutations.

Our current cryo-EM structure reveals that, in the absence of orthosteric ligands, vilazodone binds at the S1 site. Yet, it is important to integrate these findings with our previous observations of vilazodone allosteric binding ^45^. Here, the addition of imipramine revealed a vilazodone binding site with an allosteric potency of 14 nM, suggesting that imipramine may stabilize SERT in a conformation favorable for high-affinity allosteric binding of vilazodone. We hypothesize that the same effect is plausible if mutations are performed which destabilize vilazodone binding within the S1 site. In these cases, vilazodone binding would be biased towards the previously described allosteric site^45^. Furthermore, the Hill coefficient observed in [^3^H]5-HT uptake assays suggest the existence of multiple binding sites, which contrasts with our current structural findings. Our cryo-EM structure was captured in the absence of 5-HT. Therefore, the dynamic nature of 5-HT transport and its S1-binding could potentially enable vilazodone to bind both the S1 and the allosteric site. This is further corroborated by the apparent non-competitive inhibition of 5-HT transport by vilazodone ^45^.

In addition to this, we find that vilazodone can inhibit functional SERT mutants such as Y95F, I172M, S438T, and F556A, as well as double mutants involving S1 and allosteric sites with a potency similar to WT. This could be attributable to its bimodal interactions with SERT but also to its size and therefore multiple interacting residues. However, two S1 SERT mutants (A169I and I172M-S438T) reduced vilazodone’s inhibition potency, providing *in vitro* confirmation of the S1 site being the primary vilazodone binding site when other orthosteric ligands are absent.

When we investigated the ion-dependent nature of vilazodone binding, we found that vilazodone binds with equal affinities in the presence of Na^+^ and K^+^. The experiments with SERT encoding Anap suggested that vilazodone may bind to SERT conformations promoted by either Na^+^ or K^+^. These findings align with previous attempts using SERT-expressing membranes (5.8 nM and 36.7 nM, in Na^+^ and K^+^, respectively)^64^. Indeed, the reported pharmaco-chaperoning effects of vilazodone^64,65^, were proposed to stabilize inward-facing states, thereby substantiating our Na^+^-independent binding results.

To better understand the structure-activity relationships underlying its high affinity we deconstructed vilazodone into fragments. Using this approach, we demonstrated that the indole derivatives of vilazodone are potent SERT inhibitors. Addition of the piperazine group enhanced the affinity of the indole derivatives. In contrast, all the benzofuran-piperazine derivatives exhibited a low inhibition potency for 5-HT. We previously showed that the benzofuran moiety is important when vilazodone occupies the allosteric site^45^, suggesting a preference for these derivatives towards this site. This is in agreement with previous studies, in which similar benzofuran ligands showed low affinity for SERT^67^. Overall, only CDV-3–1 (indole + piperazine) showed a high affinity (2.2 nM), a high Hill coefficient and a Na^+^ and K^+^ conformational response similar to that of vilazodone in the SERT-Anap experiments. Therefore, the high affinity of vilazodone for SERT, its potential Na^+^-independence for binding and its high Hill coefficient may be attributed to the combination of the terminal indole attached to the piperazine moiety. Further investigation is necessary to fully understand the ion-independence of these different fragments. Designing multimodal ligands like vilazodone may offer improved pharmacological profiles and the potential use in treatment-resistant patients^68^.

## Methods

### Site-directed mutagenesis

The gene for full-length hSERT was encoded with a C-terminal thrombin cleavage site followed by a Twin-Strep tag and a His12-tag, each separated by GGS linkers. The construct was synthesized (Eurofins Genomics). hSERT for uptake experiments was cloned into the pUbi1z vector using the NotI and XbaI. The primers (EurofinsGenomics) were designed as follows: Y95F: gcccaggtccactgc; R104K: cctgggcaatgtctggaaattcccctacatatgttaccag; A169I: ggttatgccatctgcatcattatattttacattgcttcctactacaac; I172M: gcatcattgccttttacatggcttcctactacaac; N177V: gccttttacattgcttcctactacgtgacc; Q332A: gatagatgcagccgctgcaatcttcttctctcttggtccg; F335A: cagccgctcagatcttcgcgtctcttggtccgggc; F341V: ctctcttggtccgggcgtgggggtcctgct; S438T: caagcctgcssscgttgtgtccaagcc; E493N: ccccgtggcatactcgttcagcagcttcac; E494Q: ggtgaagctgctggagcaatatgccacggggc; Y495A: gaagctgctggaggaggcagccacggggcc; F556A: cctgttcatcatttgcagtgcgctgatgagcccgcc; Y579A: ctggagyayacatcttgggtgcgtgcataggaacctcatctttc. Cloning procedures for V86Anap and F556Anap can be found at Nygaard et al. (2024)^54^. Gene sequences were verified by DNA sequencing (Eurofins Genomics).

### [^3^H]5-HT uptake experiments

Uptake experiments were performed at room temperature (RT) using 5-[1,2-^3^H] hydroxytryptamine ([^3^H]5-HT, 43,1 Ci/mmol, Perkin Elmer) on COS-7 cells. COS-7 cells were transiently transfected with SERT WT or mutants, using Lipo2000 transfection protocol (Invitrogen): 0.8 μg SERT plasmid and 2.4 μL Lipofectamine were mixed each with 200 μL Opti-Mem^R^ (1X) and incubated for 20 minutes (min) for complex formation. The mixture was added to 13 mL DMEM 1885 medium (in house) containing 2 million COS-7 cells and seeded in a 24-well plate. The seeded cell number was adjusted to achieve an uptake level of maximally 10% of the total added [^3^H]5-HT. After 5 h, 600 μL of DMEM1885 with Penicillin, Streptomycin and L-glutamine was added. Transfections with SERT encoding Anap were performed as described previously^54^. Briefly, pre-seeded HEK293 cells were transfected at ~50 % confluency with 442 ng DNA and 1.33 μg polyethyleneimine per well in 24-well plates. A 2:2:1 plasmid ratio of SERT (pcDNA3), Anap-specific tRNA and aaRS (pANAP) and eukaryotic release factor E55D (pCAG) was used. The medium was supplemented with 15 μM Anap during and following transfection. The uptake assays were carried out 2 days after transfection. Just prior to the experiment, the cells were washed once in 400 μL uptake buffer (UB) (25 mM HEPES, 130 mM NaCl, 5.4 mM KCl, 1.2 mM CaCl_2_, 1.2 mM MgSO_4_, 1 mM L-ascorbic acid, 5 mM D-glucose, pH 7.4) RT. 50 μL of vilazodone was added to cells in the indicated concentrations, 30 min prior to the addition of 50 μL 12.25 nM [^3^H]5-HT. Nonspecific binding was determined with 1 μM paroxetine (Sigma-Aldrich). After 3 min of incubation (2–4 min for the Anap mutants), the uptake reaction was stopped by washing twice with 500 μL ice-cold UB. Cells were lysed in 250 μl 1% SDS and incubated for 60 min at 37 °C. All samples were transferred to 24-well counting plates (Perkin Elmer, Waltham, MA), 500 μL (of Optiphase Hi Safe 3 scintillation fluid (Perkin Elmer) was added, and the plates were counted in a Wallac Tri-Lux β-scintillation counter (Perkin Elmer). All experiments were carried out in triplicate.

### SERT expression and membrane preparation

The human SERT construct used for the cryo-EM studies was the full-length WT transporter tagged with a twin-strep tag followed by a His12-tag, into a pEG BacMam vector. SERT was expressed in mammalian cells using the Bac-to-Bac baculovirus expression system. Baculovirus for the expression of SERT was produced using Sf9 cells (Expression Systems). The virus was used for the infection of mammalian Expi293F cells (Gibco)^69^. The Expi293F cells were incubated at 37 °C, 5% CO2, 70% humidity, 130 rpm, in Expi293 Expression Medium (Gibco) until they reached optimal density for the expression of SERT. The cells were infected with 1.25 % of P2 virus and 2 mM of valproic acid at a cell density 3 × 10^6^ million cells/mL of medium and incubated for 72 hours (h) at 37 °C, 5% CO2, 70% humidity, 130 rpm, before harvesting. For SERT with Anap, cells were transiently transfected as described previously^54^. In short, 6 μg polyethyleneimine and 2 μg DNA, pre-mixed in Opti-MEM, were added per mL of culture together with 15 μM Anap. A 2:2:1 ratio of the following plasmids was used: pcDNA3 encoding SERT, a plasmid containing the Anap-specific tRNA and aaRS (pANAP), and a plasmid encoding a eukaryotic release factor E55D. The cells were harvested at 6200g, washed with cold PBS (in-house), harvested at 5000g, snap-frozen and stored at −80 °C. On the day of membrane preparation, cells were resuspended into 20 mL of lysis buffer (30 mM NaHEPES pH 8, 30 mM NaCl, 5 mM KCl, 10 % sucrose, 10 μg/ml Benzamidine and 10 μg/mL Leupeptin, protease inhibitor cocktail (Sigma Aldrich), 7 mM MgCl_2_, 2 μg/mL DNase, 2 μg/mL RNase) per 10 g of cell pellet. Next, cells were lysed on ice with ultrasound probe (Branson Sonifier 250 set at 50% duty cycle and output control 5). The sample was centrifuged at 1000 × g for 5 min at 4 °C to remove non-lysed cells. Supernatant was collected and the pellet was resuspended in 20 mL of fresh lysis buffer like previously. Sonication and harvest were repeated to collect the supernatant. The membranes from the resultant supernatant were harvested for 1 h at 37,200 rpm at 4 °C. Membranes were washed twice with wash buffer (30 mM NaHEPES pH 8, 30 mM NaCl, 1 M NaCl, 10 mM DTT), for 1 h at 37,200 rpm at 4 °C, resuspended in resuspension buffer (30 mM NaHEPES pH 8, 30 mM NaCl, 5 mM KCl, 10 % sucrose), snap-frozen in liquid N_2_ and stored at −80 °C.

### SERT purification

The membranes were subsequently resuspended in 4x resuspension buffer (80 mM Tris, pH 8.0, 600 mM NaCl, 20% glycerol, 10 μg/mL Benzamidine and 10 μg/mL Leupeptin, protease inhibitor cocktail (Sigma Aldrich), 0.5 mM TCEP), 9 mL per 1 g membrane. Cold solubilization buffer (200 mM Tris, pH 8.0, 200 mM DDM (10%), 40 mM CHS (2%)) was added (1 mL / 1 g of membrane) and incubated on gentle rotation at 4 °C for 1.5 h. The pellet was harvested at 40,000 rpm for 1 h and was discarded. The supernatant was filtered through 0.2 μm filters and diluted 2x in resuspension buffer (20 mM Tris, pH 8.0, 150 mM NaCl, 5% glycerol, 10 μg/mL Benzamidine and 10 μg/mL Leupeptin, protease inhibitor cocktail (Sigma Aldrich), 0.5 mM TCEP). The solubilized protein was purified by batch purification with single step nickel affinity purification and eluted with 20 mM Tris pH 8.0, 300 mM NaCl, 0.86 mM GDN (glycol-diosgenin), 0.5 mM TCEP, 300 mM imidazole, 5% glycerol, 10 μg/ml benzamidine, and 10 μg/mL leupeptin. The eluted sample was injected onto a Superdex ^®^ 200 Increase 10/300 GL gel filtration column (GE Healthcare) equilibrated in SEC buffer (20 mM Tris pH 8, 300 mM NaCl, 0.43 mM GDN) and fractions containing SERT (Extended Data Fig. 2) were pooled and concentrated using a Vivaspin 500 PES 50 kDa cut off spin filter (Sartorius, Cat#VS.0131). For SERT with Anap incorporated, the solubilized protein was immobilized to a HisTrap column and eluted with a linear imidazole gradient in buffer containing 20 mM TrisCl pH 8.0, 300 mM NaCl, 10 % (v/v) glycerol, 1 mM DDM, 0.2 mM CHS, and 24 μM lipids (1-palmitoyl-2-oleoyl-glycero-3-phosphocholine (POPC), 1-palmitoyl-2-oleoyl-sn-glycero-3-phosphoethanolamine (POPE), 1-palmitoyl-2-oleoyl-sn-glycero-3-phosphoglycerol (POPG)) in a 1:1:1 ratio. The same buffer was used for the subsequent size-exclusion^54^.

### Scintillation proximity assay

The activity of hSERT was verified by [^3^H] (*S*)-citalopram competition binding using scintillation proximity assay (SPA), and each assay was performed in white, clear-, flat-bottomed 96-well plates (Corning). SERT at a final concentration of 5 nM was added to a solution of 5% Copper HIS-Tag yttrium silicate (YSi) SPA beads (PerkinElmer), and 10 nM [^3^H](*S*)-citalopram (81 Ci/mmol) in sample buffer (20 mM Tris pH 8.0, 100 mM NaCl, 0.43 mM GDN). [^3^H](*S*)-citalopram binding was competed with (*S*)-citalopram in the indicated concentrations. All data points were performed in triplicate. Nonspecific binding was determined using 10 μM paroxetine. The binding experiment was incubated at room temperature for 1 h followed by overnight incubation at 4 °C to obtain equilibrium conditions. Binding activity was quantified on a MicroBeta scintillation counter (PerkinElmer). The data was plotted in GraphPad Prism 10 and fitted to Cheng-Prusoff^70^ equation for homologous competition, allowing for the extraction of the *K*_D_.

### Fab 15B8 expression and purification

The Fab 15B8 sequence cloned in a pFastBac vector was acquired from Eric Gouaux and included a His8 tag. A recombinant baculovirus was generated based on the Bac-to-Bac expression system standard protocols for protein expression in SF9 insect cells. The Sf9 cells were infected 2.5% recombinant baculovirus at a cell density of 2 million cells/mL of medium at 27 °C. The culture supernatant was collected 96 h after infection by centrifugation at 5,000 rpm for 20 min using a JLA 8.1000 rotor at 4 °C. 50 mM of pH 8 phosphate buffer was added, and precipitation was harvested at 5,000 rpm for 20 min using a JLA 8.1000 rotor at 4 °C. The 15B8 Fab was purified from Sf9 supernatant by Histrap HP 5 mL pre-packed column equilibrated in 50 mM phosphate pH 8, 300 mM NaCl, and protease inhibitor cocktail (Sigma-Aldrich). 15B8 fractions were collected after elution with 50 mM phosphate, pH 8, 300 mM NaCl, and 500 mM imidazole. The fractions were concentrated Vivaspin 20 PES 10 kDa cut off spin filter (Cytiva) to inject and buffer exchange using a Superdex ^®^ 200 Increase 10/300 GL gel filtration column (GE Healthcare) equilibrated 20 mM Tris, pH 8, 300 mM NaCl, and fractions containing 15B8 were pooled and concentrated using a Vivaspin 20 PES 10 kDa cut off spin filter (Cytiva). Samples were snap-frozen in liquid N_2_ and stored at −80 °C.

### Cryo-EM sample preparation

SERT was incubated in 100 μM of vilazodone for 30 min at RT. SERT and Fab 15B8 were added in a 1:2 molar ratio and incubated for 30 min at RT. The SERT-Fab-vilazodone complex was co-eluted using a Superose™ 6 Increase 10/300 GL gel filtration column (GE Healthcare) equilibrated 20 mM Tris pH 8, 100 mM NaCl, and 10 μM vilazodone. Fractions containing both SERT and Fab 15B8 were pooled and concentrated using a Vivaspin 20 PES 100 kDa cut off spin filter (Cytiva). A further 100 μM vilazodone was added in the concentrated sample. 3 μL of the sample was applied to a glow discharged for 15 mA for 30 s (Leica EM ACE200) UltrAufoil grid (R1.2/1.3 300 mesh, Quantifoil) (Jena Bioscience, Cat.-No.: X-201-Au300), and a Vitrobot Mark IV (FEI) was used to blot away excess sample plunge the grids into liquid ethane for sample vitrification at 100% humidity and 4 °C in an environmental chamber. Grids were stored in liquid N_2_ until data collection.

### Cryo-EM data acquisition and processing

Movies were acquired using a Titan Krios G2 (FEI) fitted with a Falcon 3EC detector operated in counting mode at 165,000×magnification (300 keV), yielding a raw pixel size of 0,832 Å per pixel, using EPU 3.2 software (Thermo Fisher). Movies were collected with a total doe of 42 e/Å^−2^ at a dose rate of 1.1 e/px/s and a defocus value range of −0.6 to −2.0 μm. The final reconstructions were obtained using CryoSPARC^71^ v.3.3.2 to v.4.4.1 as described in Extended Data Fig. 3.

### Model building and refinement.

The cryo-EM structure (PDB ID: 7LIA)^25^ of the human serotonin transporter was used as an initial model after removing residues 1–68 that did not correspond to electron density. The structure was initially docked in ChimeraX v1.8^72^ and next, manual adjustment and initial refinements were made in Coot v0.9.8.6^73^. Real-space refinement was carried out in Phenix v1.20.1–4487^74^. Vilazodone was generated in Phenix and optimized through the eLBOW package^75^ using the semi-empirical quantum mechanical method Austin Model 1 (AM1)^75^. An iterative process of refinement and model manual correction was performed until optimal stereochemistry and geometry were calculated by MolProbity^76^. The FSC curve between the refined model and half maps was computed and compared to avoid overfitting. Molecular graphics images were produced using the UCSF Chimera package from the Resource for Biocomputing, Visualization, and Informatics at the University of California, San Francisco (supported by NIH P41 RR-01081).

### Fluorescence Spectral Shift binding experiments

Covalent labeling of lysine residues of SERT was performed using the Protein Labeling Kit RED-NHS 2^nd^ Generation (cat# MO-L011; NanoTemper Technologies GmbH, Munich, Germany). In brief, frozen samples of SERT were thawed and buffer-exchanged from 20 mM Tris, 300 mM NaCl, 0.05% GDN, pH 8 to 20 mM HEPES, 150 mM KCl, 0.05% GDN, pH 7.5, using the A-column from the labeling kit (NanoTemper Technologies GmbH, Munich, Germany). Next, SERT samples were incubated at RT for 2 h or ON at 4°C. Approximate SERT concentration was 5 μΜ. SERT was labeled with a 5-fold molar excess of dye for 30 min at RT. Unreacted dye was removed from labeled SERT by performing SEC using Superdex 200 SEC column on an AKTA Pure FPLC system (Cytiva), with 20 mM HEPES, 200 mM NaCl, and 0.005% GDN as an elution buffer. Fractions were pulled according to 280 nm absorption and dye fluorescence profile. The labelling degree was calculated based on the SEC chromatogram at 3.7. Eluted SERT was stored at 4°C until further use. For the equilibrium experiments, 2 nM of SERT was incubated in 20 mM HEPES, 0.05% GDN, 0.4% DMSO, and 150 mM NaCl or 200 mM KCl and the tested ligand (vilazodone, (*S*)-citalopram and paroxetine) in 16 serial-dilution concentrations from 0.012 nM to 400 nM. Samples were equilibrated for 2 h at RT to reach equilibrium and measurements were recorded using Dianthus (NanoTemper Technologies GmbH, Munich, Germany). Dianthus records fluorescence emission intensities at 670 nm and 650 nm upon excitation at 590 nm. Spectral Shift is registered as a ratio of these intensities.

The $K_{d}$ is estimated by fitting the equation (1):

(1) $$f(c)=Unbound\;+(Bound\;-\;Unbound)x\frac{C\;+\;Ctarget\;+\;Kd\sqrt{(C\;+\;Ctarget\;+\;Kd{)}^{2}\;-\;4C\;Ctarget}}{2Ctarget}$$

Where f(c) is the signal at a given ligand concentration C,Unbound is the ratio signal of the target alone, Bound is the ratio signal from the complex, Kd is the dissociation constant or binding affinity and Ctarget is the final concentration of target in the experiment.

### Anap fluorescence-based experiments

For the equilibrium measurements, fluorescence emission spectra for V86Anap and F556Anap were recorded as described previously^54^. Briefly, SERT was centrifuged for 10 min at 4°C and diluted to 10 or 4 nM of V86Anap or F556Anap, respectively, in 20 mM TrisCl, pH 8, 10 % v/v glycerol, 500 μM TCEP, 1 mM DDM, 0.2 mM CHS, 24 μM lipids (POPC, POPE, POPG; molar stoichiometry 1:1:1), supplemented with 200 mM NaCl or KCl and with or without 200 nM vilazodone, CDV-3–1, or CDV-3–2. Samples were incubated for 30 min at 19 °C shielded from light before fluorescence emission spectra (excitation at 360 nm; emission at 375–550 nm) were recorded on a FluoroMax-4 spectrofluorometer (HORIBA Scientific). Spectra obtained in the absence of protein were subtracted from those obtained in the presence of protein. Of note, DMSO from the ligand stocks was diluted to ≤ 0.02 %. Control spectra for 5 nM free Anap, incubated under the same conditions as those for the SERT mutants, were measured in parallel. Experiments were repeated with protein from ≥2 transfections and purifications.

The real-time fluorescence measurements were recorded on a FluoroMax-4 spectrofluorometer (HORIBA Scientific) at 19 °C using a 5 × 5 mm quartz cuvette (Hellma). Samples were excited at 360 nm and emission was measured at 450 nm, using 9 nm excitation- and emission slit widths. Fluorescence intensities were measured as reference-corrected signals (S1c/R1c) with 0.5 sec time increments and 0.2 sec integration time. SERT F556Anap was centrifuged for 10 min at 4°C and diluted to 15 nM in fluorescence buffer supplemented with 200 mM NaCl. Following equilibration in NaCl, samples were mixed 1:1 with buffer with or without 200 nM vilazodone or imipramine (100 nM finale concentration) upon initiation of the fluorescence measurements. To correct for photobleaching, the fluorescence traces obtained without ligand were subtracted from those obtained with vilazodone or imipramine. These data were normalized to the initial fluorescence (at time 0) and fitted by a one-phase exponential model. Experiments were repeated with protein from ≥2 transfections and purifications.

### Chemistry

Vilazodone fragments (CDV-2–29, CDV-2–26, CDV-2–32, CDV-2–81, CDV-3–2, and CDV-3–1) were prepared by adapting procedures found in the literature^77–79^. Experimental details and characterization data for all compounds, including intermediates, are provided in the Supplementary Information. p*K*a calculations for vilazodone and the fragments were performed using the Maestro software tool (Schrödinger Release 2020–3: Schrödinger, LLC, New York, NY, 2020), p*K*a was calculated using Epik^63^.

### Statistical analysis

All data were plotted and analyzed using GraphPad Prism 10.1. All data are presented as mean ± S.E.M., visualized as either error bars or error envelopes. Affinities derived from logarithmic analyses are expressed as mean [S.E.M. interval]. To account for the logarithmic nature of IC_50_, statistical analyses were conducted on log-transformed values, ensuring normalized distribution and robustness in comparisons. Statistical analyses involving comparisons of more than two means were performed using one-way ANOVA with Dunnett or Tukey multiple comparison correction (95% CI, significance set at p < 0.05). All experiments were repeated at least three times using cells or protein samples from at least two independent preparations.

## Extended Data

## Data Availability

The cryo-EM reconstruction has been deposited in the Protein Data Bank under the accession code 9HCO and the corresponding EM map has been deposited in the Electron Microscopy Data Bank under the accession code EMD-52050. All data generated in this study are provided in the Extended Data, Supplementary Data and Source Data. All data, as well as the associated metadata, that support the findings in this study are also available on request from the corresponding author.
